# Supplementary material for: Macrophages Cytokine Spp1 Increases Growth of Prostate Intraepithelial Neoplasia to Promote Prostate Tumor Progression
Source: Int J Mol Sci. 2022 Apr 12;23(8):4247. doi: 10.3390/ijms23084247 (PMC9027984; doi:10.3390/ijms23084247)
Supplement: Supplementary file 1 [file ijms-23-04247-s001.zip › ijms-1663672-supplementary.pdf]

Supplemental Figure S1

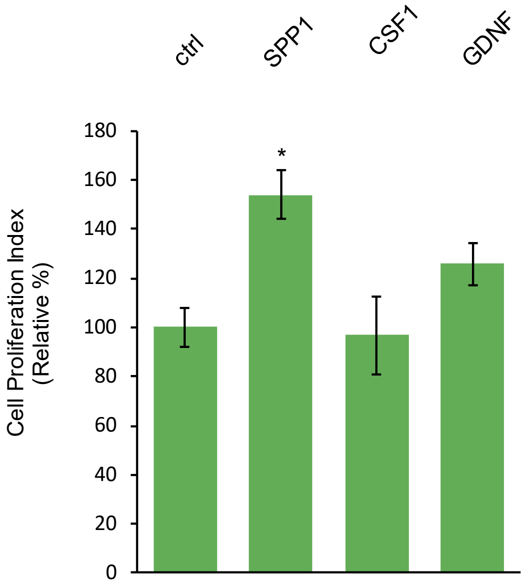

Supplemental Figure S1. Effect of recombinant Spp1, CSF-1 and GDNF on cell proliferation of Pr111 PIN cells. Pr111 PIN cells were cultured in 3D and treated with either control (ddH_2O), recombiant Spp1 (250 ng/mL), CSF-1 (100 ng/mL) or GDNF (50 ng/mL) for 72 h. At the end, cells were fixed on 3D and immunostained with cyclin D1 as well as DAPI. The ration of nuclear cyclinD1-positive cells over total DAPI-positive cells was calculated and indicated as the cell proliferation index. *: $p<0.05$ as compared to control (ddH_2O).

Supplemental Figure S2

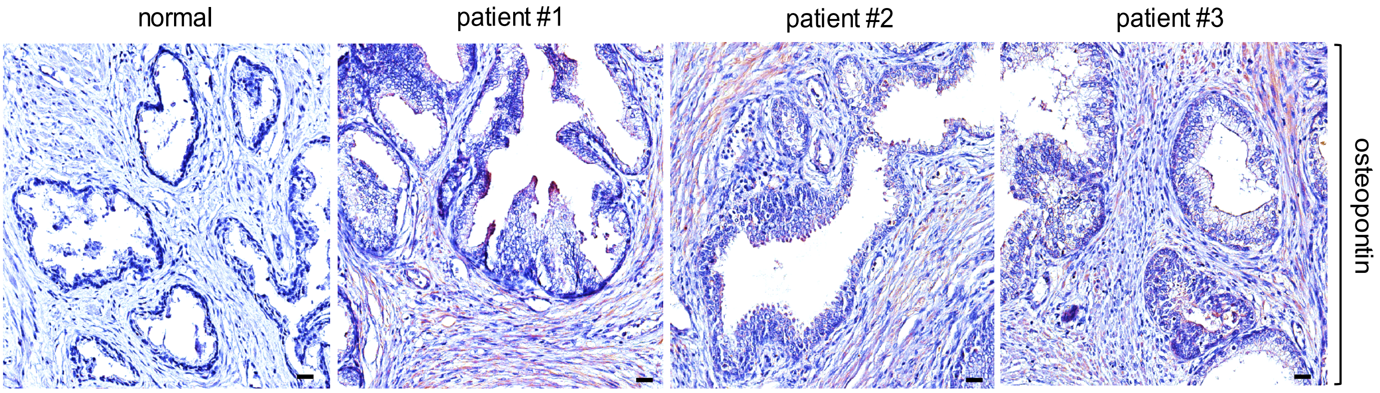

Supplemental Figure S2. Osteopontin expression in the human tissue samples of normal prostate and prostatic intraepithelial neoplasia (PIN). Human normal prostate and tissue samples containing PIN were immunostained with osteopontin to assess osteopontin expression. Scale bar: 25 μm.

Supplemental Figure S3

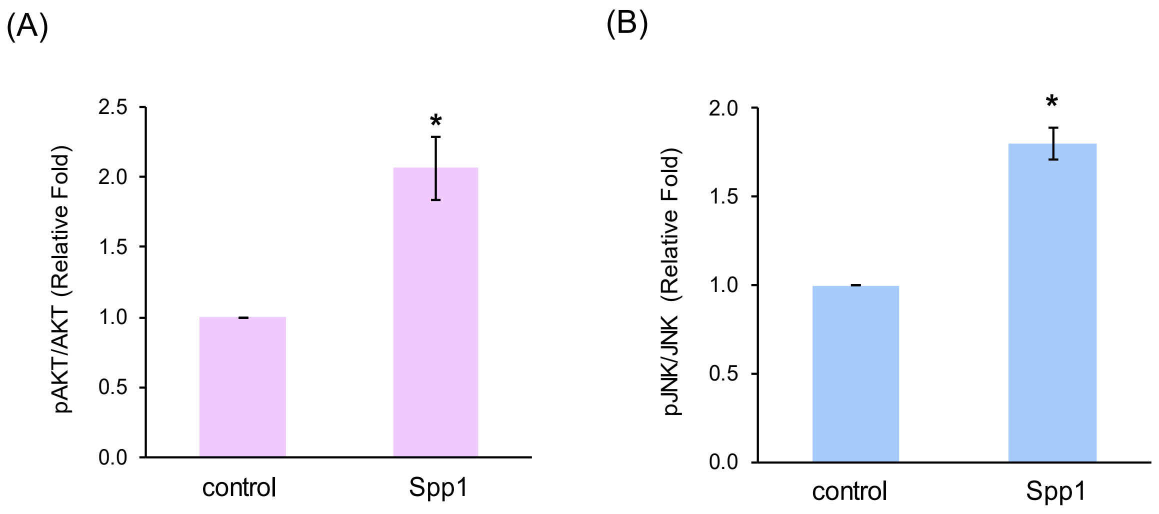

Supplemental Figure S3. Quantifcation of phosphorylated AKT and JNK levels of Pr111 PIN cells treated with or without recombinant Spp1. Pr111 PIN cells were cultured in 3D and treated with either control (ddH_2O) or recombiant Spp1 (250 ng/mL) as indicated in Fig 4. The intensity of immunoblots of pAKT, AKT (A), pJNK and JNK (B) from Fig 4A and 4B was quantified and graphed. The result shown here is from 4 independent experiments.

name	catalog number	vendor
Akt	2920	Cell Signaling Technology
Akt pS473	4060	Cell Signaling Technology
C206	ab64693	Abcam
caspase 3	9665	Cell Signaling Technology
CD38	NBP2-25250	Novus Biologicals
CD44	103001-BL	BioLegend
CD68	M087601-2	Dako
cyclin D1	55506	Cell Signaling Technology
ERK1/2	4695	Cell Signaling Technology
F4/80	MA9-1124	Invitrogen
GAPDH	sc-166574	Santa Cruz BioTech
iNOS	ab129372	Abcam
integrin αv	27096-1-AP	ProteinTech
integrin αvβ3	MAB3050	R&D System
integrin β1	12594-1-AP	ProteinTech
integrin β3	PIPA579539	Invitrogen
IκBα	4814	Cell Signaling Technology
JNK	9252	Cell Signaling Technology
p38MAPK	8690	Cell Signaling Technology
PARP	9542	Cell Signaling Technology
pERK1/2	sc-7383	Santa Cruz BioTech
pJNK	4668	Cell Signaling Technology
pp38MAPK	4511	Cell Signaling Technology
pSrc	6943	Cell Signaling Technology
Src	2109	Cell Signaling Technology
Ym1	60130	Stemcell Technology

Figure S4: Information for antibodies used in this study
